# Cholesterol metabolism and colorectal cancer: the plot thickens

**DOI:** 10.1038/s41420-023-01784-5

**Published:** 2024-01-04

**Authors:** Paulina Rzasa, Alessandro Rufini

**Affiliations:** 1 Leicester Cancer Research Centre, University of Leicester, Leicester, UK; 2 Dipartimento di Bioscienze, University of Milan, Milan, Italy

**Keywords:** Cancer metabolism, Translational research

Two main subtypes of colorectal cancer (CRC) exist; one subtype displays active wingless (Wnt) signaling through inactivating mutations targeting the Wnt negative regulator adenomatous polyposis coli (*APC*). The other subtype originates from sessile serrated adenomas and is enriched in activating mutations of the V-raf murine sarcoma viral oncogene homolog B (*BRAF*) oncogene, whereby a valine to glutamate substitution at codon 600 causes constitutive activation of the BRAF kinase and the downstream mitogen-activated protein kinase (MAPK) pathway [1–3].

Cholesterol biosynthesis through the mevalonate pathway is associated with an increased risk of developing CRC [4]. Tumorigenesis in mice carrying mutations in the *Apc* gene (*Apc*^*min*^) displays augmented cholesterol biosynthesis and is reversed by genetic or pharmacologic inhibition of the pathway [5]. Evidence suggests that mutant *BRAF* regulates the transcription factor sterol regulatory element binding protein-1 (SREBP1), a master regulator of cholesterol and lipid metabolism, in several malignancies, including CRC [6]. Nevertheless, whether cholesterol metabolism contributes to *BRAF*-mutant serrated neoplasia has not been thoroughly investigated. Recently, we identified increased expression of the mevalonate and cholesterol metabolism signature in datasets of human *BRAF*-mutant and/or serrated neoplasias as compared to normal tissues or CRCs harboring a wild-type *BRAF* oncogene. Using a mouse model carrying inducible expression of *Braf*^*V600E*^ in the intestinal epithelium, we confirmed transcriptional activation of the same signature, which was reversed by pharmacologic inhibition of the MAPK pathway [7]. Moreover, inhibition of the mevalonate pathway with statins, a class of drugs widely prescribed in the treatment of cardiovascular conditions, which inhibit the rate-limiting enzyme 3-hydroxy-3-methylglutaryl-CoA (HMG-CoA) reductase, prevented the establishment of hyperplastic crypts in the intestine of *Braf*^*V600E*^-mutant mice [7]. Thus, even in the context of serrated CRC, cholesterol biosynthesis is increased and pro-tumorigenic. However, since a subset of serrated lesions does not harbor mutations in the *BRAF* oncogene, the dependence of the signature’s expression on the MAPK pathway should be further investigated. The mevalonate pathway also synthesizes isoprenoids, fueling the biosynthesis of Coenzyme Q and protein farnesylation. Currently, it is unclear whether the anti-cancer efficacy of statins depends on their impact on the biosynthesis of sterols and/or isoprenoids [4, 5, 8]. Moreover, when comparing the expression of the cholesterol gene signature between *BRAF* wild-type adenomas and normal tissues, we did not observe enrichment in the neoplastic specimens, suggesting that transcriptional regulation might play a lesser role in the activation of cholesterol metabolism in wnt-driven CRC.

An interesting observation distinguishes serrated and non-serrated neoplasia. During Wnt-driven tumorigenesis in *Apc*^*min*^ mice, sterols act as mitogens and stimulate the proliferation of intestinal stem cells (ISCs) without affecting survival [5]. In contrast, in *BRAF*-mutant intestinal epithelium, we observed that cholesterol biosynthesis protects crypt cells from apoptosis without affecting proliferation [7]. This result, which agrees with known anti-apoptotic properties of statins [9], might highlight biological differences between crypt cells with constitutively activated Wnt or MAPK pathways, which could shape the role of ISCs in tumor progression. Indeed, whereas Wnt-driven tumors originate from *LGR5+* canonical ISCs, serrated lesions likely develop through the dedifferentiation of intestinal cells [2]. In addition, serrated CRCs encompass a population of *LGR5*-negative cancer cells characterized by a fetal-like gene signature driven by the Hippo-pathway effectors YAP/TAZ [2, 10, 11]. We have shown that the expression of *Braf*^*V600E*^ suffices to enrich the fetal gene signature in an MAPK-dependent fashion [7]. However, whether cholesterol metabolism contributes to the establishment of this subtype of fetal-like cells remains to be investigated.

Finally, what is the clinical relevance of these observations? Can statin treatment reduce the incidence of *BRAF*-mutant CRC? Although statin administration reverses crypt hyperplasia in *BRAF*-mutant mice, we could not formally demonstrate an anti-tumor impact of statins in this subtype of CRC. This may be related to the inherent limitations of the *Braf*^*V600E*^ mouse model we employed, which displays restrained tumor burden and long latency, hindering efforts aimed at assessing a direct impact on tumor development [7, 12–14]. Assessment of the preventive efficacy of statins in mouse models with higher disease penetrance [11–14] would resolve this conundrum. However, recent data indicate that the sensitivity of CRC cell lines to statin-induced cell death relies on an intact Bone Morphogenetic Protein (BMP) pathway [15]. CRC cell lines sensitive to statins display activation of the BMP pathway. Inhibiting the BMP pathway abolishes statin-induced apoptosis in otherwise sensitive cells. More specifically, statin-sensitive cell lines (e.g., RKO, HCT116, DLD1) express the BMP-related protein suppressor of mothers against decapentaplegic (SMAD4), which is lost/mutated in resistant cells (e.g., HT29, SW480). In a follow-up epidemiological study, no association between statin use and the risk of developing CRC with a mutation in *BRAF* was reported, but statins were associated with an overall reduced risk of CRC and with a larger reduction of *SMAD4*-positive CRC [16]. We confirmed these findings, showing that *BRAF*^*V600E*^ CRC cells with mutations in *SMAD4* are resistant to statin treatment in vitro [7]. Therefore, despite the fact that mutant *BRAF* drives cholesterol biosynthesis, the mutational status of *SMAD4* is likely to be a more accurate predictor of the protection conferred by the use of statins than mutated *BRAF*. Further insights into the biochemical scenarios linking cholesterol metabolism with CRC may help refine the targeted treatment of this disease.
